# Medical Items and News of Interest to the Physician

**Published:** 1885-01

**Authors:** 


### Medical Items and News,

### For the Use of Physicians.

CULLED FROM THE STANDARD MEDICAL JOURNALS OF THE WORLD.

NOTE.—These formulæ are intended only for the regular practitioner of medicine, and should be employed only by him, or under his immediate supervision.

**Scabies.**

Zinci Oxidi, 40 parts.
Bals. Peruv, 20 parts.
Mucilag. Acaciæ.
Glycerini, aa, 30 parts.
M.

**Cold Abscesses.**

Verneuil, after emptying the abscess by aspiration, injects a five per cent solution of iodoform in ether. He reports good results and no unfavorable symptoms. He has injected as much as ten ounces of the fluid into a single abscess cavity.

**Corns, Bunions and Chilblains.**

℞
Cosmoline, ℥ j.
Salicylic acid, ʒ j.

Mix by thoroughly triturating. Sig.—Apply at night and morning, and cover with soft cotton cloth.—*Medical Summary.*

**Hope's Mixture.**

This mixture is one commonly employed in the Southern States for simple diarrhœa.

℞
Aquæ camphor, ℥ iv.
Tinct. opii, gtts. xl.
Acid nitricum, gtts. iv. M.

Sig.—Tablespoonful every three hours.—*Medical Student.*

**Hay Asthma.**

Dr. M. D. O'Connell has treated hay asthma successfully (*British Medical Journal*) by the introduction into each nostril of a small piece of cotton-wool saturated with glycerine. He says that usually in from ten to fifteen minutes great relief is felt, but the wool should be allowed to remain for one hour. It is well known that the introduction of the glycerine plug into the vagina is frequently productive of great benefit in uterial congestions by the profuse watery discharge which follows its introduction and he attributes its good effects in hay fever to the same action.

**Dressing for Joints.**

As a fixed dressing for joints, etc,, requiring rest, Dr. Levis uses the following, which is painted over roller bandages or cloth, applied to the part:

Glue, lb. j.
Oxide of zinc, lb. ss.
Water, Cong. ss.

**Dyspepsia with Constipation and Piles.**

℞
Tinct. nucis vom, ℥ j.
Podophyllin, gr. j.

Triturate thoroughly to dissolve. Sig.—Five drops to be taken in water before each meal. Appetite will improve and stool become natural in a few days.—*Medical Summary.*

**Douleureux.**

Prof. Nussbauer is quoted by the *Med. & Surg. Reporter* as recommending a trial of the salicylate of soda in neuralgia of the fifth nerve, before proceeding to surgical measures. He gives from two to three drachms within 24 hours, combining a small quantity of salicylic acid. He reports a number of radical cures.

**Manaca in Rheumatism.**

Dr. C. M. Cauldwell reports having had good success in the treatment of certain forms of rheumatism by fluid extract of manaca.

The effect on man, in health, of the drug—as produced by doses of twenty drops of the fluid extract, administered five times daily for a week—was merely an increase of appetite and a "valerian-like" odor of the urine.

The most satisfactory results were obtained in subacute rheumatism, in which there was little or no rise in temperature.—*Medical Record.*

**Ointment of Salicylic Acid.**

Balmano Squire says that ointment made by *dissolving* their ingredients in *hot* lard are far more efficacious than when they are merely *mixed* with *cold* lard. In the case of chrysophanic ointment as well as salicylic ointment, the ointment should also be well mixed with a mortar and pestle during the process of cooling. Experiments have determined that at the temperature of the water-bath, hot lard will take up thirty grains of salicylic acid to the ounce, which is about the proportion in which it is usually prescribed in cases of eczema or for antiseptic dressing.—*Pharm. Journ.*

**Treatment of Sciatica.**

Dr. Comingor of Indianapolis advises, after forced flexion under ether, that the affected limb be at once put up in plaster of Paris. He states that one week in the stiff dressing should complete the cure. He considers his method of treatment particularly adapted for cases complicated with contracture.—*Amer. Pract.*

---

**Nervous Prostration.**

℞

Phosphoric acid, dil. ℥ j.
Elix. calisaya, ℥ iv.
Elix. val. ammon, ℥ ij.
Glycerine, ℥ iij.
Sherrry wine, ad. Oj.

M. S.—One to two tablespoonfuls three or four times a day.—*Ibid.*

---

**Squamous Syphilide.**

For the local treatment of squamous syphilides, Prof. Gross recommends the following elegant prescription:

℞

Hydrarg. bichlorid, grs. iv.

Tinct. benzoini, ℥ ss.

Aqua. cologniensis, ℥ j.

Aqua rosæ. ℥ iv. M.

Sig.—Apply with sponge, and hold in contact with the skin for twenty minutes.—*Col. and Clin Record.*

---

**Tuberculosis.**

Dr. Guocchi on the theory that the bacillus of tuberculosis can be destroyed by certain substances, causes his phthisical patients to inhale vapor of iodoform from a bottle with an India-rubber tube furnished with a teat, which the patient holds in his mouth. The treatment is repeated several times a day. Cigarettes may be substituted, the iodoform being packed between two pads of wadding, as is done in camphor cigarettes. Dr. Guocchi uses the following liquor, with which he declares he has obtained remarkable results in a great number of cases:

| | Grammes | |
|---|---|---|
| Idoform, powdered....... | 1.50 | [24 grains] |
| Oil of Turpentine......... | 50 | [10 min.] |
| Nut oil ...... | 150 to 200 | [5 to 7 fl. oz.] |
| Oil of Bergamot | 2.40 | [40 min.] |
| Thymol....... | 2.40 | [40 min.] |

—*Un. Med.* and *Chem. and Drugg.*

**Infantile Eczema.**

Zinci Oxidi, 40 parts.
Hydr. Oxidi Rubri, 2 parts.
Mucilag. Acaciæ
Glycerini, aa, 20 parts.

M. Secundum artem.

---

**Freckles.**

Zinci Oxidi, 10 parts.
Bismuthi Oxychloridi, 2 parts.
Hydrarg. Perchloridi, .02—0.5 parts.
Dextrini.
Aq. destill, aa, 10 parts.
Glycerini, 15 parts.

M. Boil to a paste. If it becomes hard, a few drops of water will enable it to be spread.

---

**Gout.**

Dr. J. Mortimer Granville publishes in the *Lancel* (Aug. 10, p. 272) a prescription for the relief of gout, which he states gives satisfactory results in acute and subacute gout, relieving the pain almost immediately, reducing swellings and raising the proportion of urea in the uterine from 50 to 100 per cent. The formula he gives is as follows:

Ammonii chloridi, ʒ iv.

Potasii chloratis, ʒ ij.

Glycerini, ʒ xij.

Tincturæ Iodi, ʒ ij.

Aquæ ad, ʒ xij.

M. The dose is two tablespoonfuls every third, fourth or sixth hour.—*Pharm. Journ.*

---

**Burn Ointment.**

Lead plaster, ℥ vj.

Spirit Turpentine and Linseed Oil of each ℥ j½.

Oil Origanum pure and Tinct. Opium of each ʒ vj.

Glycerine, ʒ iv.

Melt the lead plaster, then add the other ingredients, and stir till cool. For ulcerated condition of wounds, add to above amount one-half dram acid carbolic. Apply to burns, scalds, etc., by spreading on cotton batting or cloth to the thickness of one-eight inch, renewing as often as ointment becomes dry, then scraping off only the upper layer and adding fresh ointment. No other treatment is needed until the wound is fully healed, after which no scars will be left, and immediate relief is given.—*Pharmecist and Chemist.*

**Diphtheria.**

℞

Mur. pilocarpin, gr. iss.
Pepsin, (Jensen's preferred) ʒ ss.
Muriatic acid, gtt. x.
Aqua, ℥ viij.

M.

Sig.—Teaspoonful every hour.

—*The Medical World.*

---

**Eczema Capitis.**

In the common eczemas of the head in children, so numerous in dispensary practice, after two or three thorough cleansings the daily application of the following salve nearly always suffices to obtain rapid and lasting results:

℞

Acid salicylic, gr. x.
Tinct. benz., m. xx.
Vaseline, ℥ j.

M.—Ft. Ung.

On other parts where a soft salve, which easily melts as this, is not suitable, and where a firm dressing or a drying effect is desired, the following paste should be rubbed on:

℞

Acid salicylic, gr. xix.
Vaseline, ℥ j.
Zinci oxidi, amyli, *aa* ℥ ss.

M.—Leniter terend, Fiat paste (Sic.)—*Edinburg Medical Journal.*

---

**Vaccination as a Preventive of Hydrophobia and Yellow Fever.**

While M. Pasteur claims to have more than twenty dogs which he has rendered no longer susceptible to attacks of hydrophobia by inoculating them with the virus of that disease, after it had been attenuated or diluted by passing through several monkeys and rabbits; Dr. Domingo Freire, of Rio Janeiro, claims to have discovered the specific virus of yellow fever, and after inoculating some of the lower animals with it, he had used the diluted virus for vaccinating several hundred persons, and rendering them unsusceptible to the fever. Dr. Freire represents the specific virus or cause of yellow fever to be a vegetable parasite, which he has named cryptococcus xanthogenius. By what process his discovery was made we are not yet informed; neither have we sufficient knowledge of the tests to which the 400 inoculated persons have been subjected, to establish our faith in the reliability of the claims, so confidently expressed.—*Jour. Am. Med. Ass'n.*

**Papular Eczema.**

℞

Ungt. sulph., ℥ j.

Hydrarg. ox. rub., ʒ ij.

Terebinth ven., ʒ j.

Acid sulph. (pure), gtt. xxx.

M. Sig. Wash the parts and apply sparingly twice per day.

This ointment will also cure porrigo scutulata.—R. S. GABBEY, M. D. in *Phar. Gaz.*

---

**Dyspepsia.**

The following excellent prescription is given by Dr. Alfonso in the *Medical and Surgical Reporter:*

℞

Jensen's pepsin, gr. cxcij.
Sherry Wine, ℥ viss.
Glycerine puris, ℥ iss.
Acid tartaric, gr. v.

M.

Sig.—f ʒ j, after meals.

This is three grains of the pepsin to each teaspoonful.

---

**Diarrhœa.**

In the late summer and autumn, when fruit is so abundant, any simple remedy for diarrhœa, and what is familiarly called "bowel complaint," is worth knowing and remembering. Such a one was recommended by Dr. T. E. Stellwagen several years ago in his edition of Coleman's "Dental Surgery," and more recently in the pages of one of the journals. It is simply *vinegar*, preferably pure cider vinegar. The dose is about two ounces for an adult, and should be swallowed "neat"—without admixture of water.

It may also be given to infants with excellent results. To a babe a year old a teaspoonful of moderately-diluted vinegar would be the proper dose.

Its effect is to check pain, tenesmus, and tormina at once, to relieve the chill and cramps when present, and to disseminate a feeling of warmth and comfort over the body.

Even in cases of chronic diarrhœa which have long resisted treatment, this household remedy has succeeded in checking the discharges and correcting the sub-inflammatory condition of the membrances.

We shall be glad to report any experiences, favorable, or otherwise, which our readers may have with this remedy.—*Medical and Surgical Reporter.*

**Malignant Growths.**

Dr. Vogt reports, in *Centralblatt fur Chirurgerie*, two trials of a new method of treatment. The first case was one of recurring and rapidly-growing carcinoma after amputation of the breast. Half a Pravaz syringe-full of a mixture of equal parts of alcohol and oil of turpentine was injected into the substance of the tumor. Three similar injections were made in the course of two months. At the end of that time, the tumor had shrunk into a small hard mass, which showed no signs of active growth or inflammation. After each injection there was severe pain and fever, lasting about forty-eight hours, and once an abscess was formed. The second case was one of multiple sarcoma. A similar result was obtained, the tumor nearly disappearing in five weeks.

---

**Uticaria.**

Dr. McCall Anderson publishes a lecture on this subject (*Brit. Med. Jour.*), from which we deduce the following treatment: First, find out and remove the cause. In acute cases a sharp purge is useful, especially if there be indigestion. If indigestible food is still in the stomach give an emetic. Avoid stimulating diet. In chronic cases by varying the diet we may trace the offending article of food—malt liquor, spirit, white wine, vinegar, fruit, sugar, fish, vegetables, etc. In some cases complete change in diet is not of the slightest avail. When no cause is apparent, or the disease continuing after its removal, we must treat empirically. Most is, perhaps, to be expected here from atropia 1-100 gr. subcutaneously at night, or night and morning), and bromide of potash (gr. x three times a day). Continue till physiological effects are apparent. Occasionally a continuous current twice a day is useful, the positive pole being placed at the top the negative at the bottom of the spine. We may also try sulphuric ether, 20-40 drop doses, or quinine in full doses, or arsenic. Complete change of air, scene and occupation may become necessary, and a visit to Vichy is sometimes advantageous. Relief is obtained by sponging with vinegar and water, cologne, or ℞. Acidi carbolici cryst., ʒ ij; glycerini (Price) ʒ vj; eau de cologne, ℥ j; aquæ destillatæ ℥ iv; or ℞. Chloralis hydratis, camphoræ, aa ℥ ss; misce et adde glycerini (Price) ʒ j, unguenti simplicis ad, ℥ j, or tarry preparations, as a lotion of equal parts of tar, soft soap and rectified spirit; the last may exceptionally yield permanent benefit.—*Maryland Med. Jour.*

**Diphtheria.**

Dr. J. E. Marsk (*Eclectic Medical Journal*) says he has treated one hundred cases of diphtheria with a triturate of iodide of arsenic—one part of the iodide of arsenic to twenty of sugar of milk—without a death,* and in every case the whole treatment has been: ℞. Tinct. of Acon. (root or leaf ?) gtt. 5 to 10; triturate of iodide of arsenic, grs. 3 to 10; water, ℥ 4. M. S. Teaspoonful every hour. Arsenic poisoning is seldom seen; when it does appear, stop the remedy or reduce the dose. Gargle the throat with a solution of common salt in water, frequently.

---

**Chancres and Old Sores.**

Soft and hard chancres are successfully treated by applying the following mixture:

℞

Salicylic acid,
Iodoform, aa, ʒ ss.
Cannabis indica, (solid) gr. x.
Surgical collodion, ℥ i.

M.

Collodion will dissolve salicylic acid readily, and iodoform slowly. In using this, shake well and apply with a small brush, immediately to the chancre or sore. It dries immediately and completely protects the part. This will effectually destroy the specific character of the sore, and a kindly acting healing process, is set up at once. A slight burning sensation is experienced when we make the application, but this is soon followed by a cool, agreeable sensation; and when we examine the part carefully, and come to handle it, we find that a condition of partial anæsthesia exists. The application should be repeated as often as it is thrown off, which will be every two or three days. This is not only a convenient plan of treatment, but it is generally successful.—*Am. Medical Journal.*

---

**Cocaine as an Anæsthetic in Ophthalmic Practice.**

Dr. Karl Koller read a paper on this subject before the K. K. Gesellschaft der Aerzte, in Wien, on October 11th. He had already read a paper on, and made some experiments with, cocaine, at the meeting of the German Ophthalmological Society in Heidelberg, in September. It is known that the application of cocaine to the mucous membrane of the tongue causes anæsthesia of that organ, and Koller has now

*From the treatment?—ED. BISTOURY.

shown, by a series of experiments on animals, that the cornea and conjunctiva are also anæsthetized. The experiments with it in the human eye gave the following results:

1. Within from one to two minutes after the instillation of a few drops of a two per cent. solution of cocaine, the cornea and conjunctiva became anæsthetized. There was complete anæsthesia, lasting from seven to ten minutes, after which the normal sensibility was regained.

2. Within fifteen to twenty minutes after the instillation mydriasis set in, never maximal, which reached its limit in an hour, began to disappear in about two hours, and in a few hours entirely disappeared. During the mydriasis the pupil alwayr reacts promptly to light, and with it comes and goes an inconsiderable paresis of accommodation.

3. The palpebral conjunctiva becomes anæmic.

4. When the instillation was repeated every five minutes for from one-half to one hour the action of the drug on the deeper parts of the eye was seen; painful sensations from strong pressure on the ball were sensibly diminished.

5. Cocaine never causes phenomena of irritation.

Therapeutic experiments with cocaine were made in Prof. Von Reuss's Clinic, and it was seen: *a.* That it may be used as a narcotic in painful and photophobic affections of the cornea and conjunctiva, the action being limited to a few hours. It is also of service in cases in which it is necessary to touch the conjunctiva with nitrate of silver. Koller recommends its use in iritis.

*b.* That it may be used as an anæsthetic in operations on the eyes. In this capacity it was used with good results in operations for the removal of foreign bodies from the eye, in tattooing the cornea, and in operations for pterygium. Koller also recommended that it be tried in cases in which it is necessary to cauterize ulcers of the cornea with the hot iron, in puncture of the cornea and discission of cataract, in operations for staphyloma, and in iridectomies and linear extraction of cataracts; for these purposes a five per cent. solution should be used every five minutes for half an hour before the operation.—*Wiener Med. Presse.*

---

**Yellow Fever Inoculations.**

The following appeared in the Paris papers of November 11, and may be news to your readers:

In yesterday's session of the Academy of Science, M. Bouley, Vice-President, entertained his colleagues with a discovery of great importance. Since 1880 M. Domingas Freire, Professor in the Medical School at Rio Janeiro, has occupied himself with the question of yellow fever inoculation. He has even made several communications on the subject to the Academy of Medicine. He has not been able to demonstrate the microbe of yellow fever, but ascertained that the virus, of whatever nature it may be, had been attenuated, and that guinea pigs had acquired immunity.

Since then, a Frenchman, M. Rabourgeon, a pupil of Chauveau, Pouchet, and Pasteur, had been called by the Emperor Dom Pedro, to found a veterinary school at Rio Janerio. He started supplied with all the necessary apparatus for the study and culture of microbes.

Domingas Freire and Rabourgeon then united their efforts to solve the question. After having carefully experimented on guinea-pigs with the attenuated virus, they inoculated themselves as well as several students of medicine and employes of the Museum at Rio. They underwent the symptoms of mild yellow fever which dissappeared in three days.

The emperor Dom Pedro visited the laboratory, and having satisfied himself of the excellent results of their method, authorized them to experiment on human beings. Nearly 200 persons, most of whom were wharf-laborers, submitted to the vaccination and remained unaffected, while around them were large numbers of their comrades succumbing to the disease.

English sea-captains sailing in these latitudes, learning that yellow fever was epidemic at Rio Janeiro, had all their crews vaccinated, first setting the good example themselves.

The description of the microbe will eventually be published. Meanwhile it is certain that the attenuated virus has preserved about 500 animals and human beings, who had submitted to the vaccination.

These happy results must be compared with the report made by Dr. Rochard, that of twenty-five physicians sent to Senegal to care for the yellow fever patients, twenty-three had soon died, and the conclusion is near that this vaccination is destined to save the Europeans who visit the places where yellow fever is endemic. We are happy to see, adds one of the Paris newspapers, that one of our countrymen, a pupil of our own savants, is among the new benefactors of mankind.—*Ther. Gaz.*

**Eczema.**

STARCH PASTES.

Useful in eczema. In this case, the property of drying must be imparted to the paste by the addition of oxide of zinc, sulphur, etc.

Zinci Oxidi, 50 parts.
Acid Salicylici, 2 parts.
Amyli Oryzæ, 15 parts.
Glycerini, 15 parts.
Aq. destillat, 75 parts.

Mix simultaneously, and heat until reduced to 140 parts.

A similar paste for acne consists of:

Sulphuris præcip, 40 parts.
Calcii Carbonat, 2 parts.
Zinci Oxidi, 20 parts.
Amyli Oryzæ, 15 parts.
Glycerini, 20 parts.
Aq. destillat, 75 parts.

M. Reduce by boiling to 120 parts.

DEXTRIN PASTES.

Zinzi Oxidi, 40 parts.
Dextrini (pulverized),
Aq. destillat, aa, 20 parts.
Glycerini, 40 parts.
Sulphuris sublim, 2 parts.

M. Boil to a paste.

**Koch's Latest Researches on Cholera.**

Koch has two great qualities as an investigator—unequalled *technique*, and powers of patient observation. His experience has been vast, and each piece of work which he has done has been remarkable in leaving but little to correct, either by himself or subsequent observers. No writer on mycology is more reliable; not one has proved himself worthier of professional confidence.

Doubts have arisen in the minds of many, concerning the cholera bacillus, on account of the observations of Lewis, and of Finkler and Prior; and upon these we are glad to have Koch's criticisms, which have just appeared in the *Deutsche medicinische Wochenschrift*.

Lewis (*Lancet*, September 20, 1884) states that a bacillus resembling that of cholera can be found in the mouth, but Koch shows, in a very few words, that this form has been known for some years, that it differs from the comma form of cholera in being longer, more slender, and not so blunt at the ends; and it further differs in this all-important particular, that it will not develop in the weak alkaline peptongelatine, in which alone the cholera bacillus can be cultivated.

His criticisms on the work of Finkler and Prior, on cholera nostras, show how necsssary it is for men to have a proper preliminary training before undertaking such investigations. From their own statements he easily proves that they could not possibly have obtained pure cultivations. They appear, also, to have been so far astray as to mistake the part of the bacillus which all observers regard as the spores. After considerable difficulty Koch obtained some of their culture material, and found in it four different microbes, of which one resembled slightly the commabacillus, but is larger and plumper, and in its mode of growth quite different, growing much more rapidly in gelatine or on potato, and showing unmistakable differences in the form assumed in the cultures. It is a totally distinct microorganism, and very probably has no special connection with cholera nostras. The culture which Finkler and Prior made was from stools which were not quite fresh, but they had preparations from fresh stools which were believed to show the comma-bacillus, but those which Koch examined contained only the ordinary intestinal forms.

Three cases of undoubted cholera nostras have since been examined by Koch, and neither in the stools nor in the intestines could comma-bacilli be found, nor did they develop in cultivations.

He remarks, in conclusion, that the experiments of Rietsch and Nicati, on the production of cholera in animals, have been successfully repeated in the Berlin Hygienic Laboratory. The material of a pure cultivation was so far diluted that the quantity injected did not contain more than the hundredth part of a drop. When placed in the duodenum the animals died in from one and a half to three days. The mucosa of the small intestine was reddened, the contents wattery, and the comma-baccilli were found in extraordinary numbers. The condition was similar to that of a recent human case. The exceedingly small amount injected precludes the possibility of an intoxication produced by the action of any poisonous product.

The latest observations of Koch afford additional strong confirmatory evidence of the correctness of his views concerning the etiology of cholera and its connection with the comma-bacillus.

**Squills Mixture.**

℞

Tinct. squills, ♏ v.

Wine of ipecac, ♏ iv.

Surup of tolu, ♏ xx.

Water, s. q., ʒ j.

M.—Dose, ʒ j for child 1 year old.

---

**Anti-Syphilitic Mixtures.**

℞

Bichlorid. mercury, gr. $\frac{1}{32}$.

Iodide potassium, grs. x.

Comp. syr. sarsap, ʒ ss.

Water, s. q., ʒ ij.

M.—Dose, ʒ ij.

IODIDE POTASSIUM.

℞

Iodide potassium, grs. x.

Comp. syr. sarsap, ♏ xv.

Water, s. q., ʒ j.

M.—Dose, ʒ j.

---

**Compound Syrup of Wild Cherry.**

Noticing of late several inquiries for a comp. syr. wild cherry, I inclose the following, which is always dispensed in the vicinity of Charleston when comp. spr. wild cherry is ordered:

℞

Morph. acet, gr. iij.

Tr. sanguin, ʒ ij.

Vini antimon, ʒ iij.

Syr. pruni virg, ℥ iij.

Vini ipecac, ʒ iij.

M.

Some druggists filter the resulting compound; others prefer to dispense it unfiltered.
—G. A. OTGEN, *in New Remedies.*

---

**Sea-sickness.**

T. T. Reynolds, the surgeon of a transatlantic steamer, speaks favorably of the use of drop doses of the liq. atropiæ sulphatis (Ph. B.) in a teaspoonful of water, and given hourly until the physiological effects of the drug appear. This is serviceable only when used early in the malady, and may be accompanied by drachm doses of equal parts of brandy and iced water, or iced lemonade when there is dryness of throat from th effects of the atropine.

Speaking of the use of the bromides in large doses (90 grains per diem), as recommended by the late Dr. G. M. Beard, Dr. Reynolds mentions several instances in which much harm has resulted; delirium, followed by impotence and incapacity for business of considerable duration, being the most notable results.—*Drug Circular.*

**Dysentery.**

℞

Tinct. kino, ♏ xx.

Tinct. catechu, ♏ xx.

Tinct. opium, ♏ xv.

Tinct. capsicnm, ♏ v.

M.—Dose, ʒ j (in water.)

---

**Diarhœa Mixtures.**

℞

Nitric acid, ♏ xx.

Tinct. opium, ♏ viiss.

Tinct. camphor, ♏ viiss.

Comp. syr, rhubarb, ʒ j,

Syr. ginger, ʒ j.

Water, s. q., ℥ j.

M.—Dose, ℥ j.

---

**Rheumatic and other Pains.**

COMP. CHLOROFORM LINIMENT.

℞
Chlorof. commercial, }
Tinct. opium, } of each ℥ j.
Tinct. camph. }
Tinct. capsic, ℥ ss.
M.—External use.

LINIMENT STIMULANT.

℞

Ammonia water, ʒ ss.

Tinct. capsicum, ʒ ss.

Soap liniment, ʒ j.

M.—External use.

---

**Typhoid Fever.**

M. Vulpian (Medical Press) gives his preference to salicyiic acid, as compared with the sulphate of quinine in typhoid fever. He holds it to be not only antithermic, but also antipyretic, although we are unable to fully understand the difference between those two terms which is implied by his use of them. He has never found it to produce any deleterious action on the heart, and has remarked the absence of bed sores in cases in which it has been employed. We presume he has reference to large doses of salicylic acid in those cases, for we think the experience of practitioners is very unanimous on not only the uselessness, but postive injuriousness of small doses, at least of the sulphate of quinine in typhoid fever. This drug is permissible in this disease only as an antipyretic, for which it must be given in very large doses. In our experience, however, this is beneficial rather in preventing the rise of temperature after it reduction by means of cold baths, than in directly reducing it *per se,*

**Alkaline Laxative.**

℞

Chloride sodium, ʒ j.

Bicarb, soda ʒ iv.

Carbon calcium, ʒ iss.

Sulph. soda, ℥ iss.

M.—Dose, ʒ ij.

---

**Diuretic.**

℞
Corrosive sublim, gr. $\frac{1}{32}$.
Powd. squills, gr. j.
Powd. digitalis, gr. j.
M.—Make 1 pill.

ANOTHER.

℞
Ext. hyoscyamus, 3 grs.
Oxide zinc, 2 grs.
Honey, s. q.
M.—Make 1 pill.

---

**Intermittent Fever.**

The untoward effects of quinine have, ever since the earliest introduction of this alkaloid, afforded reasons for hoping that some other agent equally efficacious and devoid of danger might be introduced to take its place. Many articles from time to time have been introduced, but after their thorough trial they have either been abandoned entirely, or referred to the secondary list of the physician who has to do with intermittent fever. As bearing upon this point, of the ability of drugs other than quinine to break up intermittent fever, we refer to the remarks recently made by Dr. Beardsley, before the Chirurgical Society of Montreal. Dr. Beardsley refers to his experience, running through fifty years, in the treatment of intermittent fever without quinine. The treatment which he adopted may be epitomized as follows: Give first a brisk alterative purgative, such as a combination of aloes, blue mass and capsicum, made into pill form. Calomel may be substituted for blue mass. This he followed, as soon as it had induced a free operation of the bowels, with an aromatic bitter, combined perhaps with an alkali or in combination with boneset tea, drunk freely. The boneset tea was an element in the cure of his cases which he would not have overlooked. He stated that this method had been very satisfactory in his and others' hands, and was also successful in many cases where quinine had utterly failed, even after it had been thoroughly pushed, to the extent of 20-grain doses three times a day, or even oftener.

**Diuretics.**

℞

Fl. ex. buchu, ʒ ss.

Tinct. hyoscyam, ʒ ss.

Sol. citrat. potass, ʒ j.

M.—Dose, ʒ ij.

DIGITALIS.

℞

Infus. digitalis, ʒ j

Acet. potass, grs. xv.

Water, s. q., ʒ ij.

M.—Dose, ʒ ij.

---

**Sore Throat.**

℞
Biborate soda, ʒ j.
Sat. sol. chlorate potass, ℥ j.
Water, s. q., ℥ viij.
M.—Use as a gargle.

CHLORIDE ZINC GARGLE.

℞
Chloride zinc, grs. x.
Glycerine, ℥ ss.
Water, s, q , ℥ iv.
M.—Use as a gargle.

TANNIN AND ALUM GARGLE.

℞

Alum, ʒ ss.

Tannin, ʒ ss.

Glycerine, ℥ ss.

Water, s. q., ℥ iv.

---

**Malarial Hæmaturia.**

At the recent meeting of the Mississippi State Medical Association, Dr. J. E. Halbert read a paper, in which he stated that he had had a large experience in the treatment of this formidable malady. He sums up his course of treatment as follows: "I first advise prophylaxis; purgatives of calomel in large doses, encouraged by enemata; warm bathing to promote action of the skin; sinapisms to epigastrium—ice, and, if the patient is weak, champagne; carbolic acid, in small doses; digitalis, as a gentle diuretic and heart stimulant; as astringents, gallic acid and ergotine, if the hemorrhage is continuous and exhaustive. In addition, I allow lemonade and bitartrate of potash *adlibitum*, but the sheet.anchor is quinine hypodermically. Nourish by rectum, if necessary, and avoid, if possible, giving medicines, or anything except ice, champagne, and concentrated nourishment by stomach."

